# High-throughput morphology mapping of self-assembling ternary polymer blends†

**DOI:** 10.1039/d0ra08491c

**Published:** 2020-11-24

**Authors:** Kristof Toth, Chinedum O. Osuji, Kevin G. Yager, Gregory S. Doerk

**Affiliations:** Department of Chemical and Environmental Engineering, Yale University New Haven Connecticut 06520 USA; Department of Chemical and Biomolecular Engineering, University of Pennsylvania Philadelphia Pennsylvania 19104 USA; Center for Functional Nanomaterials, Brookhaven National Laboratory Upton New York 11973 USA gdoerk@bnl.gov

## Abstract

Multicomponent blending is a convenient yet powerful approach to rationally control the material structure, morphology, and functional properties in solution-deposited films of block copolymers and other self-assembling nanomaterials. However, progress in understanding the structural and morphological dependencies on blend composition is hampered by the time and labor required to synthesize and characterize a large number of discrete samples. Here, we report a new method to systematically explore a wide composition space in ternary blends. Specifically, the blend composition space is divided into gradient segments deposited sequentially on a single wafer by a new gradient electrospray deposition tool, and characterized using high-throughput grazing-incidence small-angle X-ray scattering. This method is applied to the creation of a ternary morphology diagram for a cylinder-forming polystyrene-*block*-poly(methyl methacrylate) (PS-*b*-PMMA) block copolymer blended with PS and PMMA homopolymers. Using “wet brush” homopolymers of very low molecular weight (∼1 kg mol^−1^), we identify well-demarcated composition regions comprising highly ordered cylinder, lamellae, and sphere morphologies, as well as a disordered phase at high homopolymer mass fractions. The exquisite granularity afforded by this approach also helps to uncover systematic dependencies among self-assembled morphology, topological grain size, and domain period as functions of homopolymer mass fraction and PS : PMMA ratio. These results highlight the significant advantages afforded by blending low molecular weight homopolymers for block copolymer self-assembly. Meanwhile, the high-throughput, combinatorial approach to investigating nanomaterial blends introduced here dramatically reduces the time required to explore complex process parameter spaces and is a natural complement to recent advances in autonomous X-ray characterization.

## Abstract 2

Compositionally graded electrospray deposition combined with grazing incidence small angle X-ray scattering forms a high-throughput approach for mapping phase behavior in ternary mixtures as demonstrated here using block copolymer blends.

## Graphical abstract

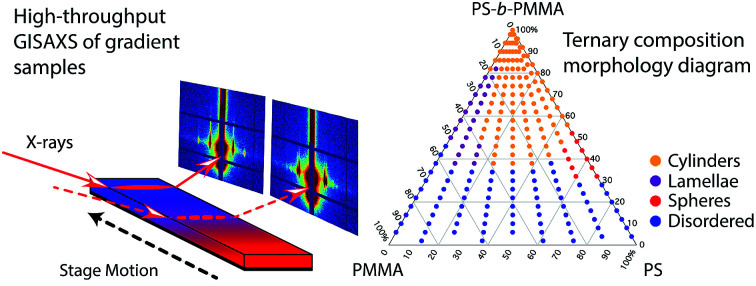

## Introduction

Block copolymers (BCPs) have been intensively studied based on their ability to form various morphologies with periodic and uniform nanoscale domains (*e.g.* cylindrical, lamellar, spherical, gyroid) as a result of microphase separation of covalently bonded polymer “blocks”;^1^ these morphologies have been applied to lithographic nanopatterning,^2–5^ synthesis of isoporous separation membranes,^6–9^ creation of optical metamaterials,^10^ and surface nanotexturing for antireflection of light.^11,12^ While BCP self-assembly on its own engenders new paths for engineering materials, blending BCPs with homopolymers, molecules, nanoparticles, or other BCPs is a powerful approach to augment phase behavior in ways tailored to specific needs.^13^ For example, blending offers ways to tune domain size and spacing,^14,15^ guide self-assembly towards desirable and novel morphologies,^16^ direct domains to form nonregular device-oriented geometries,^17^ promote vertical domain-orientation,^18,19^ accelerate ordering kinetics,^20–23^ enable template-directed selection between coexisting morphologies,^24^ and control pore size in BCP membranes.^25,26^

The recent work by some of our authors highlights opportunities for materials synthesis *via* polymer self-assembly afforded using a particular category of blends: ternary blends of a diblock copolymer with very low molecular weight homopolymers (≤∼3 kg mol^−1^) that are chemically equivalent to each block. When the degree of polymerization of each homopolymer (*N*_H_) is much lower the than that of the polymer block in which it resides (*e.g. N*_H_ ≪ *N*_A_ or *N*_B_ for a A-*b*-B diblock copolymer), the homopolymers act as plasticizers that distribute uniformly within each domain,^27^ and thereby thoroughly wet the “brush” composed of corresponding diblock chains.^28^ These homopolymers at the “wet brush” limit dramatically accelerate self-assembly kinetics, thereby achieving a level of domain order that is effectively impossible using the neat BCP.^20–22^ The uniform distribution of wet brush homopolymers throughout the domains also increases the area per BCP chain at the domain interfaces, allowing the BCP chains to relax from their normally stretched configuration, resulting in a negligible change or decrease in the self-assembled domain spacing with increasing homopolymer fraction.^14,21,22,29^ This is in contrast to more well-studied blends with higher molecular weight homopolymers, where homopolymer localization at domain centers leads to substantial increases in domain widths and spacings.^14,15,30–35^ The combination of enhanced ordering kinetics with reduced domain spacing makes blending of wet brush homopolymers especially appealing for nanolithographic applications, motivated by decreased dimensions required by continued device scaling^3,5^ or patterned magnetic media.^36^

Blending is also a powerful approach to rationally control self-assembled nanodomain morphology,^35,37–41^ in some cases obtaining ordered morphologies that are inaccessible using neat (unblended) BCPs.^13,42–46^ Considering the advantages of using wet brush homopolymers, however, investigations of their morphological behavior in ternary blends with BCPs have been relatively rare.^14,29,35^ Even in blends with larger molecular weight homopolymers, a large majority of previous investigations have been restricted to binary blends^33–35,39,43,47^ or ternary blends at a single fixed ratio between two blended homopolymers,^14,15,32,48^ in part due to the laborious and time-intensive requirements of sample preparation and characterization. Unfortunately, this leaves substantial gaps in knowledge for large regions of blended composition space, ultimately precluding a detailed understanding of compositional trends in the morphology, structure, and functional properties of blends.

Here we report a new high-throughput method to map nanostructural material properties of solution-deposited blend systems across a complete ternary composition space with a high degree of granularity. In this method, a recently introduced electrospray deposition (ESD) instrument^49^ is used to prepare gradient composition “libraries”, and the compositionally varying nanoscale structure within the blend is subsequently characterized by synchrotron grazing-incidence small-angle X-ray scattering (GISAXS). We apply this method to generate a morphology diagram for a self-assembling ternary blend consisting of a 67 kg mol^−1^ PS-*b*-PMMA BCP that forms PMMA cylinders with 1.1 kg mol^−1^ wet brush PS and PMMA homopolymers, collecting more than 220 measurements at distinct compositions over a time period of approximately a couple days. Composition regions are identified for a disordered phase as well as extremely well ordered cylindrical and spherical and lamellar morphologies. Notably, ordered lamellae identified in the BCP/PMMA binary wet brush blends investigated here (one axis of the ternary morphology diagram), have not been demonstrated previously in comparable blends with higher molecular weight homopolymers.^33^ Moreover, the high granularity of our approach permits not only clear definition of morphology transitions, but also systematic quantification of domain spacing with respect to composition and the identification of compositions at which long-range order is maximized.

## Results and discussion

### Experimental approach

**Fig. 1:**
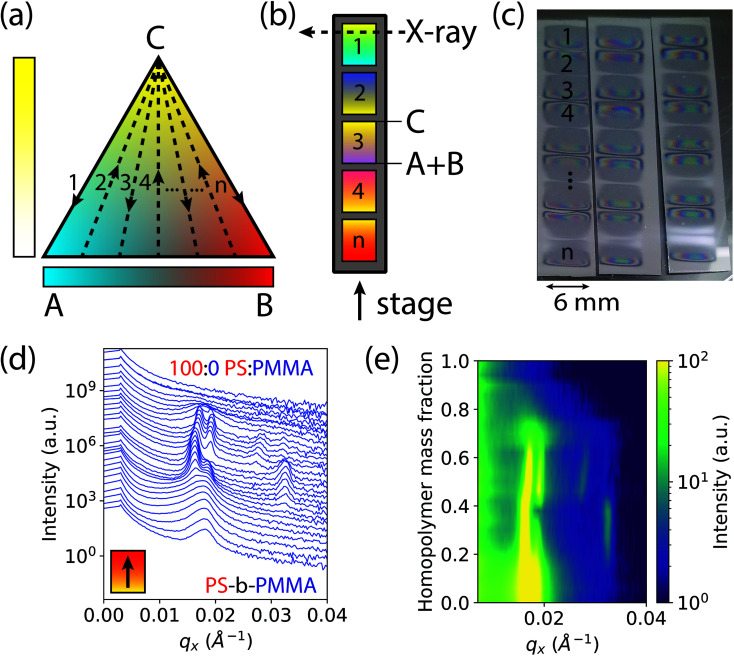
Schematic of a ternary diagram and single substrate library for GISAXS measurements. (a) Discretization of a ternary diagram through “*n*” gradient stripes shown in dashed lines. (b) Multiple gradient stripes on a single wafer substrate extending from component C to a blend of components A + B, corresponding to the dashed lines in the ternary diagram. In our case, A, B, and C correspond to 1.1 kg mol^−1^ poly(methyl methacrylate) (PMMA), 1.1 kg mol^−1^ polystyrene (PS), and 67 kg mol^−1^ cylinder forming polystyrene-*block*-poly(methyl methacrylate) (PS-*b*-PMMA), respectively. (c) Consecutively deposited gradient stripes shown on three single wafer “libraries” for three process conditions (ESD substrate temperatures). (d) Compiled waterfall plot of scattering intensity as a function of *q*_*x*_ and homopolymer mass fraction in a single gradient from PS-*b*-PMMA to pure homopolymer PS. (e) Colormap plot of the intensity as a function of *q*_*x*_ and homopolymer mass fraction, for better visualization of data in (d).

The basis of our approach is a first-of-its-kind instrument that can create binary and ternary composition libraries with continuous composition gradients by electrospray deposition (ESD).^49^ In order to analyze polymer thin films by GISAXS, the ternary composition parameter space was divided into 1-dimensional gradient segments or “stripes” that were deposited sequentially on a single wafer. A schematic ternary composition map is shown in Fig. 1a, in which the vertices of the triangle denote the respective pure components A, B, and C. While the ESD instrument is capable of rastering a full two-dimensional ternary composition space for transmission X-ray experiments,^50^ GISAXS measurements provide information for both in-plane and out-of-plane morphology orientations, a critical feature in assembling a morphology diagram for block copolymer systems. Furthermore, the larger X-ray scattering volumes in GISAXS allow for measurements of thin films without the need for additional staining or contrast enhancement, such as by replication of the domain morphology in alumina by block-selective infiltration and etching.^51^ To form each gradient stripe, the gradient syringe pumps were set up to dispense at a constant total flow rate while continuously increasing (decreasing) the flow rate of homopolymer blend (A + B) solutions and simultaneously decreasing (increasing) the flow rate of block copolymer solution (C) as the stage moved underneath. Gradient stripes were deposited on a single sample consecutively as shown in Fig. 1b. This ternary setup is helpful if trends or transitions of interest lie along composition profiles with changing concentrations of C at a constant ratio A : B. An alternative discretization of the composition space varies the fraction of A and B continuously while holding the fraction of C constant within each stripe (see ESI Fig. S1†). The deposited gradient stripes are 5 mm in length with 1 mm of space in between. The gradients alternated C to A + B, A + B to C, A + B to C, and so on, in order to maintain continuous flow of solution through the nozzle and minimize unwanted mixing. In the particular case studied in this work, the three components are 1.1 kg mol^−1^ PMMA (A), 1.1 kg mol^−1^ PS (B), and 67 kg mol^−1^ cylinder forming PS-*b*-PMMA (C). The polymer films deposited by ESD with a typical thickness of ∼300 nm were thermally annealed to promote self-assembly into ordered morphologies (see Methods section for details).

This rapid process allows the entire ternary composition parameter space to be covered on a single wafer. Three such parameter spaces are shown in Fig. 1c for three ESD conditions (varying substrate temperatures). Interestingly, in all samples the BCP starting side of each gradient stripe exhibited a cloudy film appearance while the homopolymer blend side did not. This cloudy appearance on the BCP-rich side is a result of μm-scale (lateral) surface roughness that increases diffuse optical scattering, whereas the homopolymer-rich side is substantially smoother. We attribute this difference to the more than three orders of magnitude lower viscosity of the homopolymer blend arising from the low molar mass of the homopolymers and the associated increase in free volume which reduces their glass transition temperatures (see ESI† Section S2). Assuming that polymer is deposited by ESD as approximately circular droplets that are free of solvent, or that solvent evaporates far more rapidly than the polymer droplets spread, the lower viscosity of the homopolymer blend facilitates faster polymer spreading and redistribution that in turn reduces film surface roughness. This hypothesis is corroborated by a report by Rietveld *et al.* who found that the surface roughness of continuous poly(vinylidine fluoride) films deposited by ESD is inversely proportional to the droplet shear rate and is hence proportional to droplet viscosity.^52^ Investigating the effect of homopolymer viscosity on film uniformity will be a topic of future research.

In order to accommodate the large X-ray path length through the sample in GISAXS, ESD was performed using an elongated extractor slit to deposit polymer in an area roughly 6 mm wide by 0.5 mm long at discrete compositions. There is some variance in the thickness along the path of the X-ray beam near the edges of the deposited sample as denoted by changes in its reflection colors. However, a thickness ≥ 6 cylinder layers was maintained along the beam path over a majority of the film, avoiding the effects of strong confinement.^53^ Moreover, the maximum thickness gradient along the beam direction is less than 0.01%, and therefore domain alignment effects observed for much larger thickness gradients (>1%) are not expected here.^54^

GISAXS measurements were conducted at increments of 0.2 mm along the 5 mm gradients of increasing homopolymer fraction using an approximately 0.2 mm wide X-ray beam. The sample holder positions for each measurement on the stripes were converted to a homopolymer mass fraction value, giving a 4% increment in homopolymer fraction for each measurement. Structural information was extracted from images of two-dimensional GISAXS scattering patterns by taking a line cut along the scattering vector normal to the plane-of-incidence, *q*_*x*_. These line cuts were compiled into waterfall and colormap plots of intensity as a function of *q*_*x*_ and homopolymer mass fraction, shown by example in Fig. 1d and e, respectively.

A PS-*r*-PMMA random copolymer brush with a 69% PS mole fraction had been grafted to the substrate prior to polymer deposition by ESD (see Methods section). This grafting step was performed for two reasons. First, the brush reduces the interfacial tension between the polymer film and the substrate, which is important to inhibit dewetting of the low molecular weight homopolymer during ESD or subsequent annealing.^55^ Second, a 70% PS mole fraction brush has been shown to promote robust vertical orientation for PMMA-cylinder forming PS-*b*-PMMA films well-over 100 nm thick,^56^ while vertical lamellae orientation is generally observed for brushes with PS mole fractions around 55–60%.^57,58^ Therefore, grafting a 69% PS mole fraction brush is expected to promote the vertical orientation of at least cylindrical PMMA domains, if not necessarily lamellae. However, we found that self-assembled domains in blends with high homopolymer mass fractions tend to orient in ways that directly counter these expectations, as discussed in the next section.

### Mapping a ternary composition morphological diagram

**Fig. 2:**
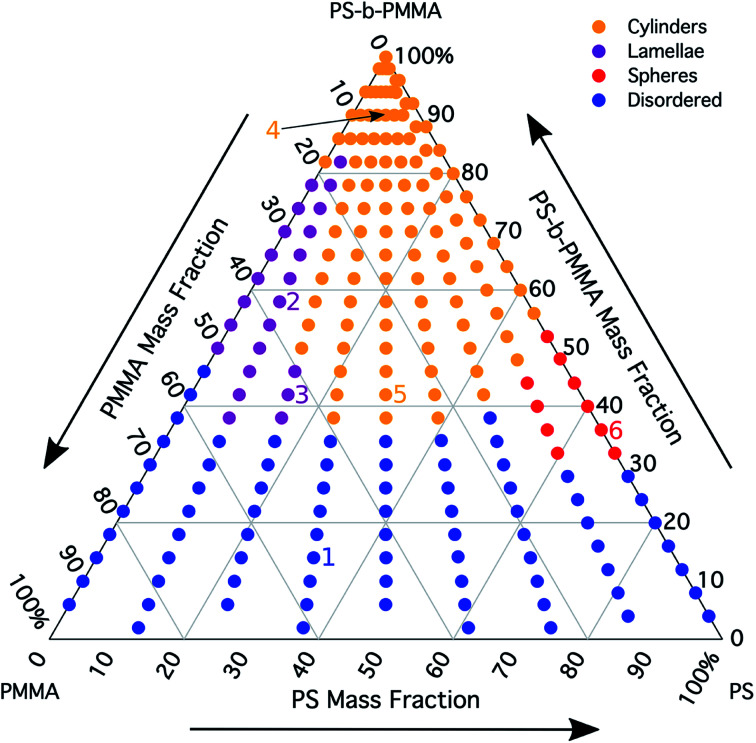
Ternary morphology diagram constructed from ESD gradient samples for 1.1 kg mol^−1^ PMMA homopolymer, 1.1 kg mol^−1^ PS homopolymer, and 67 kg mol^−1^ PS-*b*-PMMA block copolymer. Cylindrical, lamellar, spherical, and disordered morphologies are identified. The numbered points correspond to exemplary cases that are elaborated on further in the main text.

The morphology diagram for the BCP homopolymer ternary blend system derived from analysis of GISAXS data is shown in Fig. 2, in which pure BCP that natively forms a morphology of hexagonally arranged PMMA cylinders in a PS matrix is located at the top corner, whereas pure PMMA and PS homopolymers are located at the lower left and right corners, respectively. Each colored point on the diagram indicates a single GISAXS measurement. A primary peak position 
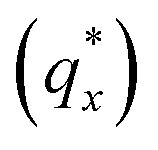
 was ascertained along the scattering vector normal to the plane of incidence (*q*_*x*_) just above the Yoneda band, and morphology at each measurement point was deduced by analyzing line cuts taken along *q*_*x*_ in the GISAXS scattering patterns. We note that coexistence between the morphologies in some composition regions may be potentially inferred from GISAXS line cuts; in such cases we have assigned the dominant morphology. A slight shift in the location of the points is apparent for the last two stripes, corresponding to PS : PMMA homopolymer ratios of 87.5 : 12.5 and 100 : 0, respectively. This shift is due to a different position correction for these two stripes, as discussed in the Experimental methods section. Line cut waterfall and color plots for each stripe are provided in Fig. S2 and S3 in the ESI.†

**Fig. 3:**
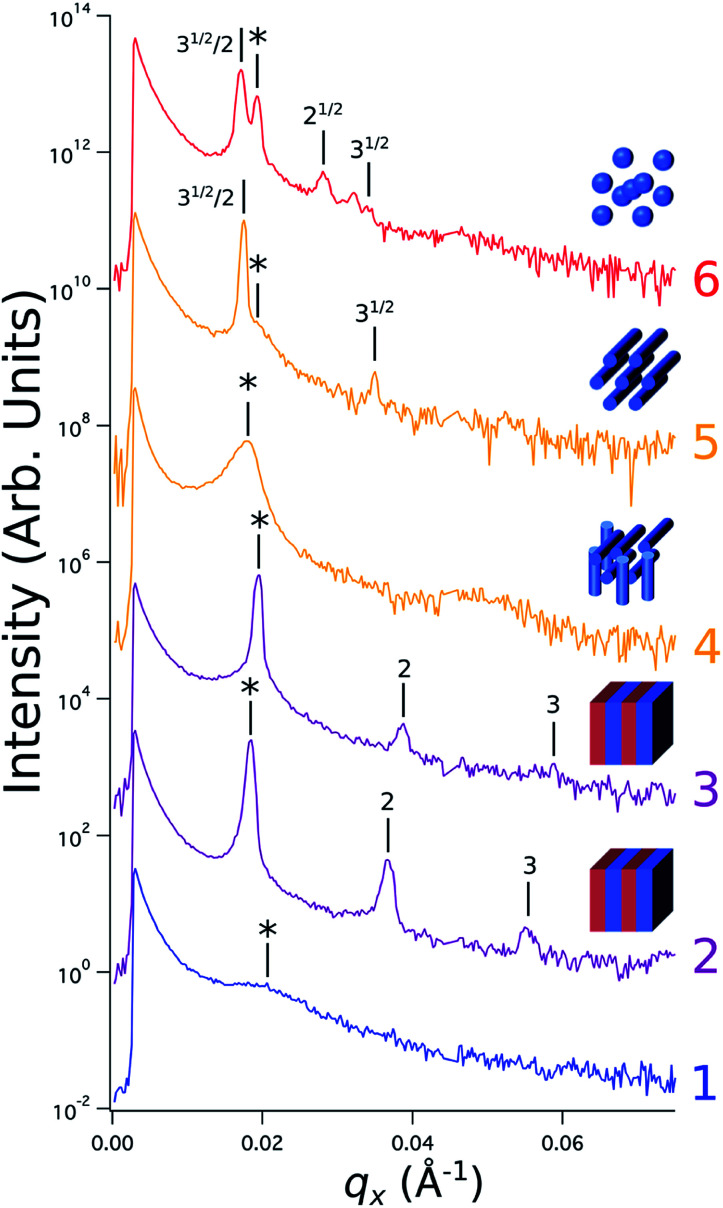
Sample line cuts in *q*_*x*_ at *q*_*z*_ = 0.026 Å^−1^ from GISAXS scattering patterns corresponding to individual points (1–6) in Fig. 2, listed to the right of the plot. Curves are shifted vertically for clarity. The first-order peak 
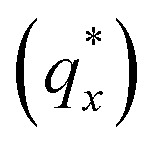
 is marked by an asterisk, and the relative positions of higher-order peaks are marked by vertical black lines. Disordered (1), lamellar (2–3), cylindrical (4–5), and spherical (6) morphologies are identified based on the *q*_*x*_ position of higher-order peaks relative to 
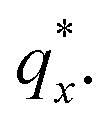
 The numbers in black text indicate the relative peak positions, and the schematics indicate the assigned morphologies.

**Fig. 4:**
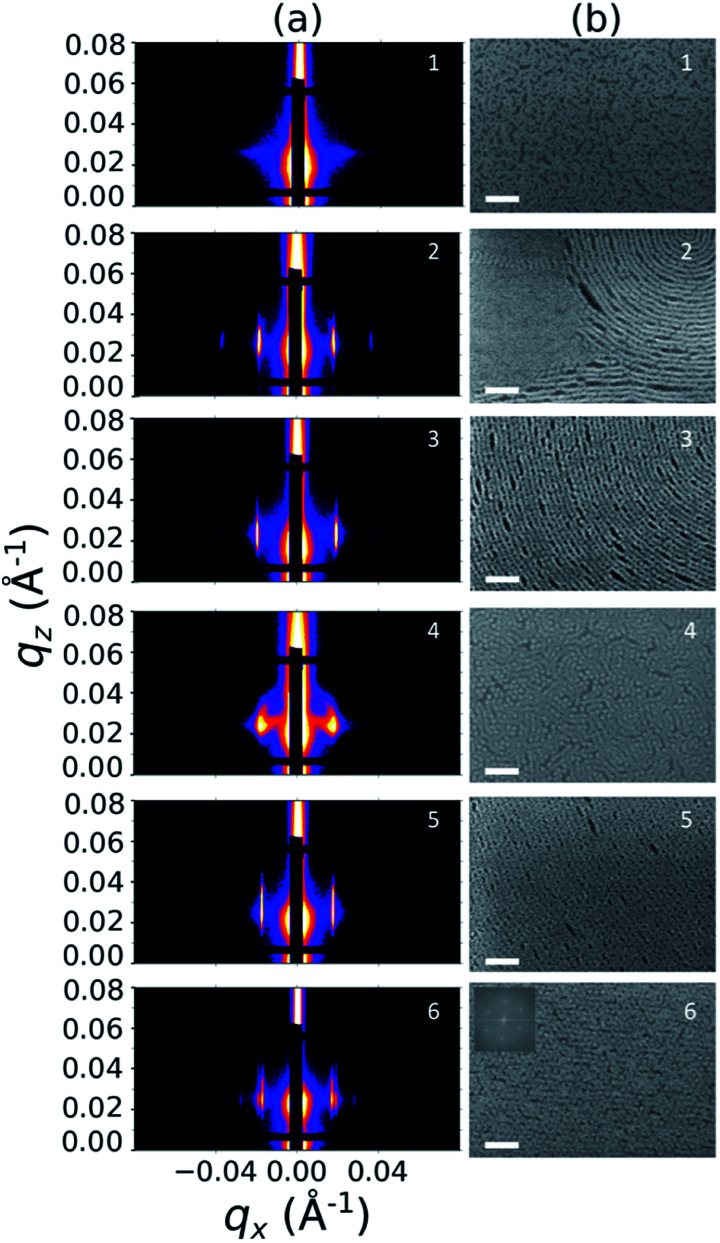
Structural characterization of selected points 1–6 in Fig. 2, including (a) GISAXS scattering patterns and (b) top view SEMs. The PMMA domains were selectively infiltrated with aluminum oxide to enhance image contrast for SEM analysis. All scale bars are 200 nm. The inset in (b) for point 6 is a Fourier power spectrum of that SEM.

Sample line cuts corresponding to numbered points in Fig. 2 are depicted in Fig. 3. To corroborate analyses of the line cuts, GISAXS scattering patterns for each numbered point in Fig. 2 are also included in Fig. 4a, while scanning electron micrographs (SEMs) taken in the vicinity of where the GISAXS data were collected on the sample are provided in Fig. 4b. To create contrast for SEMs (after GISAXS measurements), PMMA domains were selectively infiltrated with aluminum oxide using an atomic layer deposition reactor as has been described earlier,^21^ followed by partial etching using an oxygen plasma (see Methods). Note that the aluminum oxide infiltration is limited by diffusion to the top ∼100 nm of the film; as a result, bright aluminum oxide replicas of the former PMMA domains are observed in top down SEMs, while PMMA domains further down in the film may appear darker in cross-section SEMs due to the fact that PMMA etches faster than PS using an oxygen plasma.

A disordered morphology is observed at high homopolymer mass fractions, as marked by a single very broad peak in GISAXS line cuts (*e.g.* point 1 of Fig. 3). The corresponding diffuse GISAXS scattering pattern and SEM showing no clear structural motif in Fig. 4a and b, respectively, support this assessment. No signs of macrophase separation are observed. This is expected based on the segregation strength of the homopolymers, *χN*_H_, where *χ* is the Flory–Huggins interaction parameter and *N*_H_ is the geometric mean average degree of polymerization for the two homopolymers. For these two homopolymers, *χN*_H_ ≈ 0.4, which is well below the threshold of 2 necessary for macrophase separation.^59^ Therefore, microphase separated morphologies in the ternary blend system are anticipated to traverse through an order-disorder transition (ODT) with increasing homopolymer mass fractions. Inspection of Fig. 2 indicates that this ODT generally occurs a homopolymer mass fraction of ∼0.64, though it extends to a higher homopolymer mass fraction of ∼0.7 for PS-rich ternary and binary blends. On the other hand, the ODT occurs at only ∼0.52 for the BCP/PMMA binary blend, suggesting that binary blends including homopolymers that are chemically equivalent to the minority block are more prone to disorder.

**Fig. 5:**
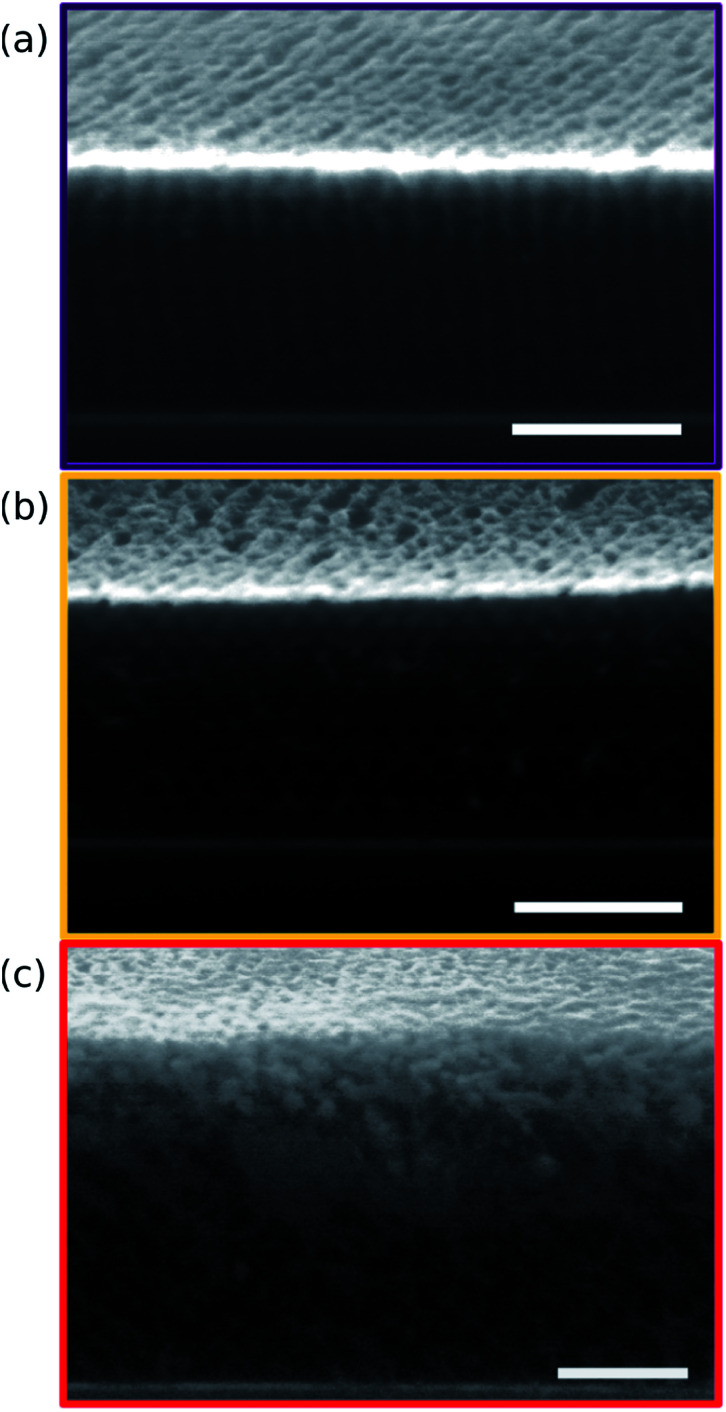
Cross-sectional SEMs taken from regions of the sample corresponding to points 3 (a), 5 (b), and 6 (c) in Fig. 2. Image contrast has been enhanced through post-processing to make the morphology apparent through the entire film thickness. PMMA domains appear bright near the top surface of the film as a result of aluminum oxide infiltration, but dark further down where aluminum oxide infiltration is incomplete. All scale bars are 200 nm.

Points 2 and 3 in Fig. 2 have been designated as lamellar morphologies, based on the integer spacing between peaks in the line cuts corresponding to these points in Fig. 3. The strong intensity of the second-order peak indicates asymmetry in the domain widths.^60^ The corresponding GISAXS scattering pattern for point 2 in Fig. 4a exhibits the trace of an isotropic ring, indicating that some portion of the lamellae are oriented randomly. A fraction of lamellae may also be oriented horizontally; lamellae in this orientation would result in a peak along the *q*_*z*_ direction that is likely hidden behind the beamstop, especially if only a small fraction of lamellae are oriented horizontally (and therefore result in a low scattering intensity). An isotropic ring is absent in the GISAXS scattering pattern in Fig. 4a corresponding to point 3, signaling that nearly all the lamellae are vertically oriented at this composition. The top view SEM corresponding to point 2 (Fig. 4b) also shows an ostensible horizontal lamellae domain alongside line patterns indicative of vertical lamellae, while the SEM for point 3 exhibits only lines. A cross-sectional SEM corresponding to the area in the vicinity of point 3 shown in Fig. 5a confirms that these vertical lamellae extend down to the substrate. The degree of order and persistence of vertical lamellae through the entire film thickness of approximately ∼8 times the self-assembled domain period is striking, especially when considering the limited thickness in which PS-*b*-PMMA lamellae were previously observed to orient vertically, even on substrates with appropriately balanced interfacial interactions.^57,61^

Assignment of the cylindrical morphology is based on combined analysis of line cuts, two-dimensional GISAXS scattering patterns, and SEMs. The line cut in Fig. 3 corresponding to point 4 in Fig. 2 (homopolymer mass fraction = 0.1) for instance shows only one strong first-order peak with a slight asymmetry in scattering intensity towards lower *q*_*x*_. The scattering pattern for this point (Fig. 4a) shows that this peak forms part of an isotropic ring that curls towards lower *q*_*x*_ with increasing *q*_*z*_, while an SEM taken from this region shows a random distribution of vertical and horizontal cylinders. Taken together, these data provide evidence that the parent BCP and blends with low homopolymer mass fractions (≲20%) self-assemble to form only poorly ordered and randomly oriented cylinders. This observation is consistent with the results of our previous study using the same neat BCP when annealed at the same temperature.^21^ It is likely that annealing at a higher temperature and for a longer time will produce cylinders with a consistent orientation,^56^ at least at the top surface if not through the entire film.^62^

On the other hand, blending in larger fractions of low molecular weight homopolymer has been shown to dramatically enhance self-assembly kinetics, producing a well-ordered morphology throughout the film.^21^ This is evident based on the GISAXS scattering data from point 5 in Fig. 2. The position of 
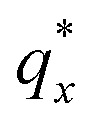
 in the line cut corresponding to this point in Fig. 3 is determined based on continuity with its position at lower homopolymer mass fractions (see ESI Fig. S2 and S3†) and is attributed to vertically oriented cylinders. A much more prominent peak centered at 
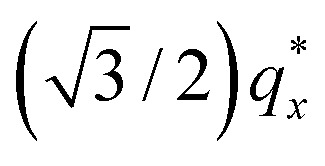
 is attributed to horizontally oriented cylinders where the lattice vector between parallel cylinders is aligned with the substrate surface.^21,63^ The dominance of this second peak at lower *q*_*x*_ shows that nearly all the cylinders are horizontally oriented in this section of the composition space, an assessment that is corroborated by the presence of a higher-order peak at 
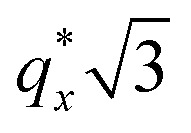
 that is a signature of hexagonal symmetry.^63^ This assignment is further supported by the corresponding top-view SEM for point 5 (Fig. 4b) and a cross-sectional SEM in the same vicinity shown in Fig. 5b. Indeed, as demonstrated in Fig. 5b, extremely well-ordered hexagonal cylinders persist without topological defects down to the substrate.

It is notable that the well-ordered hexagonal cylinder morphology observed at high homopolymer mass fractions for various PS : PMMA ratios of the blended homopolymer is consistently oriented horizontally (*i.e.* cylinders parallel with the substrate), while the best-ordered lamellae observed here orient vertically. As noted in the previous section, these results run counter to expectations with respect to domain orientation for films deposited on a PS-*r*-PMMA random copolymer brush with a 69% PS mole fraction. We infer that the interaction of the low molecular weight homopolymers with the brush plays a key role in dictating the domain orientation. Han *et al.* have pointed out that a preference for chain ends to segregate towards a hard wall (*i.e.* the substrate) favors the presence of shorter polymer chains there.^58^ This tendency may in turn result in an increased concentration of homopolymer at the substrate. As a result, an energy penalty would be expected if PMMA cylinders were oriented horizontally as neither the PMMA block nor PMMA homopolymer would be in contact with the substrate, but this penalty may be compensated for by the accumulation of PS homopolymer at the substrate and by the reduction in enthalpy induced by putting only PS in contact with the PS-rich brush. On the other hand, a horizontal lamellar morphology would impose a considerably larger energy penalty by preventing the relatively higher fraction of PMMA homopolymer in the blend from being able to concentrate at the hard substrate surface.

A cylinder-to-lamellae transition is observed for blends studied here at total PS mass fractions (the weighted addition of the BCP PS mass fraction with the mass fraction of PS in the homopolymers) ranging from 0.46 to 0.60. On the other hand, a transition to the lamellar morphology could be naïvely expected based on the mean field BCP phase diagram^1^ (after converting from volume fraction to mass fraction) at a total PS mass fraction in the range of 0.57 to 0.63. Comparison between these ranges highlights the fact that morphological transitions in blends of BCPs with homopolymers do not necessarily follow the phase behavior predicted for neat linear diblock copolymers. Rather, the morphology in blends of BCPs with homopolymers is dictated by the distribution of homopolymer within each domain.^27^

It is more appropriate then to compare the system studied here with a previous report by Jeong *et al.*, who found that up to 45% (v/v) of a 4 kg mol^−1^ PMMA homopolymer could be blended with a ∼88 kg mol^−1^ PS-*b*-PMMA BCP that forms PMMA cylinders (PMMA block volume fraction = 0.3) before macrophase separation, and observed no crossover to a lamellar morphology within this range.^33^ In other words, the total PS mass fraction could be decreased to ∼0.36 without resulting in a cylinder-to-lamellae transition. In the present study, we observe a cylinder-to-lamellae transition in the binary PS-*b*-PMMA/PMMA blend at a total PS mass fraction of ∼0.55. Thus, this blend system exhibits a cylinder-to-lamellae transition that has not been demonstrated using higher molecular weight PMMA. This behavior is consistent with the self-consistent field theory (SCFT) calculations of Matsen^27^ that show the volume fraction of homopolymer in binary blends at which a morphological (order–order) transition occurs is highly sensitive to the degree of homopolymer localization within domains, and therefore on the homopolymer molecular weight. Specifically, as homopolymer molecular weight is reduced, a higher fraction of homopolymer is driven by entropy towards domain interfaces and less of it is localized at domain centers. As a result, the system will transition to a morphology possessing a higher mean curvature towards the opposite domain in order to accommodate the increased volume of homopolymer residing at domain interfaces. The PMMA homopolymer at the wet brush limit used in this study (1.1 kg mol^−1^) has an even lower molecular weight than the shortest PMMA homopolymer (4 kg mol^−1^) in the binary PS-*b*-PMMA/PMMA blends studied by Jeong *et al.*, and is therefore expected to distribute with even higher concentrations at domain interfaces. This increased PMMA homopolymer concentration at domain interfaces in turn facilitates the observed transition to a lamellar morphology.

It is also worthwhile to note that the transition to a lamellar morphology occurs at a total PS mass fraction of ∼0.55 when only PMMA homopolymer is added, while the lamellar morphology transition occurs around a total PS mass fraction of ∼0.46 when the homopolymer is added at a 25 : 75 PS : PMMA ratio, implying that the cylinder morphology is more stable in a ternary blend than in the binary one. Phase diagrams for ternary blends calculated by Janert and Schick using SCFT have shown that a significantly higher concentrations of homopolymer can swell microphase separated structures in ternary blends of a lamellar diblock copolymer with equivalent homopolymers (both 0.3 times as long as the diblock) compared to binary blends of the same BCP with just one of the homopolymers.^41^ They attribute this behavior to the tendency of homopolymers to “relieve stress” arising from the inability of the BCP to optimally fill space. In a binary blend, stress may be relieved by homopolymer only in chemically equivalent domains without incurring an enthalpic energy penalty, while both homopolymers may act to relieve stress in ternary blends. Applying this argument to the ternary system studied here implies that blending in low molecular weight PS homopolymer that distributes throughout the PS matrix may in part counterbalance the stress on PS block chains imposed by PMMA homopolymer residing near the domain interfaces.

Previous reports have also demonstrated that a binary blends of a lamellar diblock copolymer and a homopolymer chemically equivalent to one of the blocks can stabilize ordered bicontinuous morphologies, which can be attributed to the role that the homopolymer plays in relieving packing frustration.^64^ However, no clear-cut evidence of a gyroid or another ordered bicontinuous morphology was uncovered in traversing the composition space in this work. The difference may be attributed to the very low molecular weight of the homopolymer used here in comparison to previous work. Higher molecular weight homopolymer may localize to regions that relieve packing frustration for the BCP, where any loss in translational entropy for the homopolymer is compensated by the gain in conformational entropy. However, very low molecular weight homopolymers distribute more uniformly to maximize translational entropy, resisting the localization that is necessary to relieve packing frustration. As a result, lower molecular weight homopolymers may not stabilize complex bicontinuous morphologies with respect to classic BCP morphologies (*e.g.* cylinder, lamellae). Indeed, Winey *et al.* found that while an ordered bicontinuous morphology (identified as ordered bicontinuous double diamond, but likely a gyroid morphology^64^) emerged when 30% (w/w) of 6 kg mol^−1^ or 14 kg mol^−1^ PS was blended with a 49 kg mol^−1^ lamellar polystyrene-*block*-polyisoprene (PS-*b*-PI) BCP, the lamellar morphology transitioned directly to a cylindrical one at the same homopolymer mass fraction when the PS homopolymer molar mass was reduced to 2.6 kg mol^−1^.^35^

Finally, for the 87.5 : 12.5 and 100 : 0 PS : PMMA stripes, a peak emerges at 
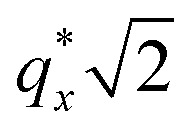
 as the homopolymer mass fraction increases, as shown by the exemplary line cut in Fig. 3 corresponding to point 6 in Fig. 2. This additional peak indicates a transition into a spherical morphology. Both the line cut and the scattering pattern corresponding to point 6 in Fig. 4a are consistent with BCC spheres.^65^ However, the origin of a peak between the 
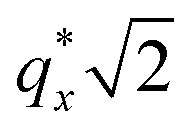
 and 
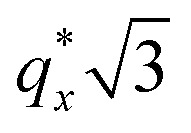
 is unclear. A top view SEM taken in the vicinity of point 6 shows a well-ordered hexagonal pattern (see also the inset Fourier transform to the SEM). A cross-sectional SEM taken from the same region (Fig. 5c) confirms that these are spheres and not vertical cylinders.

### Quantifying trends in self-assembled nanostructures

Beyond the determination of morphology diagrams, this method is designed to produce gradient composition data in formats amenable to systematic analysis of essential or functional properties of blended thin films. For self-assembling BCPs, these properties typically include the domain spacing and the extent of long-range order in the topological domain morphology. The wet brush homopolymers used here are expected increase long-range order by reducing the effective glass transition temperature of the blend,^66,67^ enhancing polymer diffusivity through reduced entanglement,^68,69^ and lowering energy barriers for defect annihilation.^20,22,70^ Moreover, as noted in the Introduction, the particular distribution of homopolymer is a key determinant of domain spacing.^27^ Therefore, it is especially important to understand how these properties are influenced by the amount of homopolymer in the blend and the relative ratio between PS and PMMA homopolymers.

**Fig. 6:**
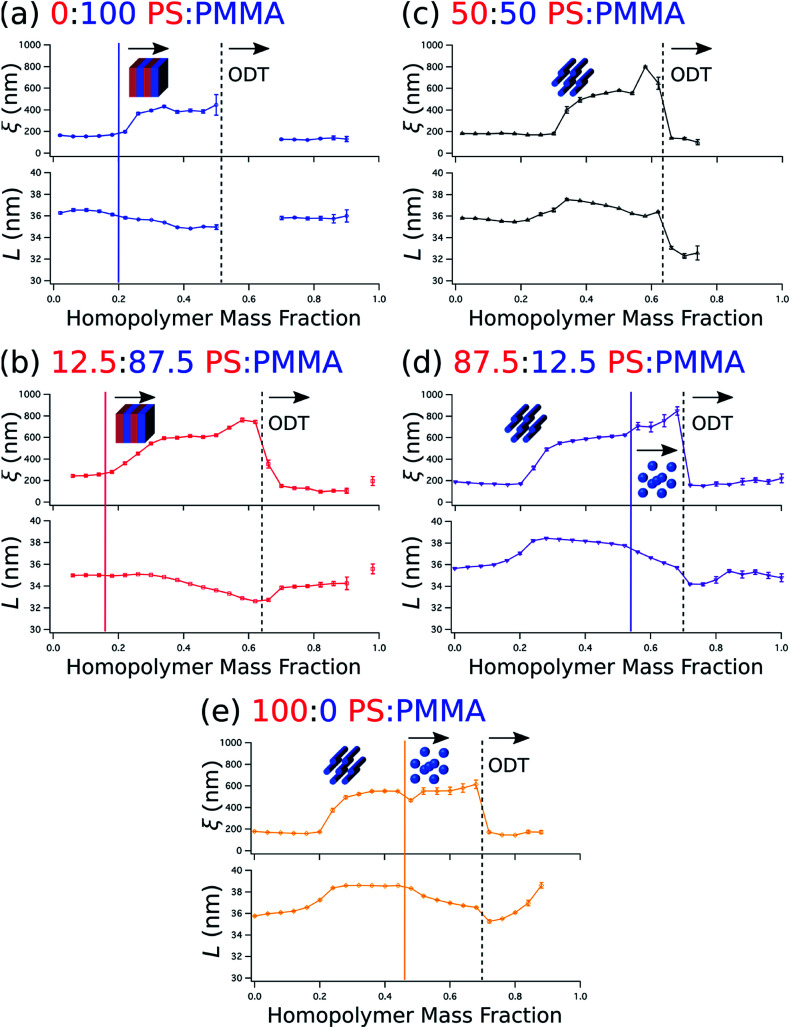
Plots of grain size (*ξ*, top) and domain spacing (*L*, bottom) against the homopolymer mass fraction for selected stripes in which homopolymer is blended in PS : PMMA mass ratios of 0 : 100 (a), 12.5 : 87.5 (b), 50 : 50 (c), 87.5 : 12.5 (d), and 100 : 0 (e). The positions of order disorder transitions (ODTs) are marked by dashed black lines, while order–order transitions are marked by solid color lines. The morphology is depicted schematically on the plots (the presumed morphology at low homopolymer mass fractions is cylindrical).

We quantify the degree of topological order by the grain size (*ξ*) extracted using a Scherrer-type analysis^71^ of the width of the first-order scattering peak in line cuts along the scattering vector normal to the plane of incidence that are obtained through Gaussian fitting. The domain spacing, or lattice period (*L*), is derived from the peak center position (*q*_*x*,*c*_) using the same fits according to the relationship that *L* = 2π/*q*_*x,c*_. Grain size and lattice period data are plotted against the homopolymer mass fraction in Fig. 6a–e, where the curves represent data from a selected stripe in which the homopolymer is blended in PS : PMMA ratios of 0 : 100, 12.5 : 87.5, 50 : 50, 87.5 : 12.5, and 100 : 0, respectively. For each stripe, the top plot displays grain size, while the bottom plot displays the lattice period. Dashed vertical black lines indicate the position of ODTs, identified by sharp drops in grain size, and solid vertical lines mark the positions in which morphologies change (*i.e.* order–order transitions). General inspection of these plots reveals important aspects about how the blended homopolymer affects the ordering of the self-assembled system. For instance, there is a clear composition threshold in the range of ∼10–30% homopolymer (w/w) below which the homopolymer has negligible effect on grain size, but above which the grain size rapidly rises to a factor of 2–3 or more than the grain size of the neat BCP. This marked improvement in domain ordering is consistent with our previous work involving blends of PS-*b*-PMMA with ∼3 kg mol^−1^ PS and PMMA homopolymers. In particular, the grain size in blend thin films with PS-*b*-PMMA lamellae increased only modestly at a homopolymer mass fraction of 30%,^22^ while the grain coarsening behavior measured by GISAXS for horizontally-oriented cylinders in 20% (w/w) blends with the same cylinder-forming BCP used here was approximately the same as for the neat BCP.^21^

It is also apparent that the grain size in the binary blend systems (Fig. 6a and e, top) remains relatively unchanged above this threshold, while in the ternary systems (Fig. 6b–d, top) the grain size continues to increase up to the ODT. Furthermore, blending only PMMA with the BCP (Fig. 6a) has a less positive effect on ordering than the opposite binary blend case of adding only PS (Fig. 6e), as evidenced by the smaller grain size and the presence of an ODT in the vicinity of a lower homopolymer mass fraction of ∼0.52 for the PMMA binary blend. Grain size also appears to increase a bit more rapidly with increasing homopolymer mass fraction for PS homopolymer-rich stripes (Fig. 6d and e, top) after the transition from a cylinder to a sphere morphology. However, these order–order transitions occur just before ODTs, where homopolymer addition appears to have an increased positive impact on ordering kinetics. Indeed, the grain size plots in Fig. 6 indicate that extremely well-ordered morphologies are formed in these binary and ternary blends with homopolymer mass fractions just prior to an observed ODT, generally in the range of 0.5 to 0.6.

The domain spacing *L* also exhibits distinctive relationships with respect to blend morphology and homopolymer composition. *L* almost immediately contracts for the BCP/PMMA binary blend (Fig. 6a, bottom), and continues to do so until it reaches an ODT. This contraction is consistent with the behavior observed in the wet brush limit described previously.^14,27,29^ The 12.5 : 87.5 PS : PMMA stripe (Fig. 6b, bottom), for which lamellae is also the most prominent morphology, exhibits similar behavior except that the decrease in lattice period does not commence with the initial addition of homopolymer. Rather, the decrease in lattice period begins at a homopolymer mass fraction that approximately coincides with passing through the composition threshold for increased grain size discussed earlier.

Sensitivity of the lattice period to homopolymer mass fraction beginning at the composition threshold for improved ordering is also observed in the 50 : 50, 87.5 : 12.5 and 100 : 0 PS : PMMA stripes that embody a cylindrical morphology (Fig. 6b–d, bottom). In these stripes the lattice period initially increases rapidly at the composition threshold. This may be explained by the emergent dominance of horizontally oriented cylinders over randomly oriented cylinders, which is concomitant with the dramatic increase in the degree of order. In this case, the measured lattice period (*L* = 2π/*q*_*x*,*c*_) using line cuts along the *q*_*x*_ direction is from the peak which arises due to horizontal cylinders and represents the distance between parallel cylinders rather than the nominal distance between rows of cylinders.^21^ This shift would give rise to an approximate apparent increase in the measured lattice period by a factor of 
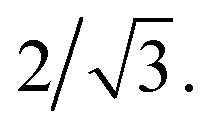


After this initial increase that coincides with the presence of highly ordered horizontal cylinders, the lattice period decreases with increasing homopolymer mass fraction up to the ODT. However, as the fraction of PS homopolymer in the blend increases, the rate of change of the lattice period with respect to homopolymer mass fraction decreases. Indeed, the lattice period for the 100 : 0 PS : PMMA stripe is largely unchanged until the transition from a cylindrical to a spherical morphology at a homopolymer mass fraction of ∼0.46. This suggests that the PMMA homopolymer, which occupies the minority cylinder domains, is largely responsible for the changes in lattice period, whereas the PS homopolymer has negligible effect while the morphology remains cylindrical. This behavior can be attributed to the much higher volume of the matrix domain (approximately 2–5 × the volume of the cylinder domains) that the PS homopolymer can occupy to maximize translational entropy, including interstices between cylinders, without having a substantial effect on the interfacial area per BCP chain. Eventually, however, enough homopolymer will reside at the domain interface and induce a change to a spherical domain morphology with a higher mean interfacial curvature. After this transition to a spherical morphology, the domain spacing decreases more rapidly with increasing homopolymer mass fraction for the stripes corresponding to 87.5 : 12.5 and 100 : 0 PS : PMMA homopolymer blend ratios.

## Experimental section

### Materials and substrate preparation

Substrates used for the gradient electrospray deposition were boron doped Si test wafers, 500 μm thick, 〈100〉 orientation, with a resistivity range of 1–100 ohm cm (University Wafers). The Si substrates were treated by grafting a hydroxyl-terminated random copolymer brush to their surfaces according to the method we have described previously.^22^ Briefly, a hydroxyl-terminated random copolymer brush of PS and PMMA provided by the Dow Chemical Company (PS-*r*-PMMA-OH; 69% styrene, determined by ^13^C NMR) was diluted to 1% (w/w) in propylene glycol monomethyl ether acetate (PGMEA) and spun onto the Si wafers at 1500 rpm. The wafers were baked at 250 °C for 5 minutes in a nitrogen-enriched environment to promote grafting of the brush to the substrate, followed by rinsing in PGMEA at 3000 rpm to remove ungrafted brush polymer. Cylinder-forming 67 kg mol^−1^ PS-*b*-PMMA (*M*_n_ = 46-*b*-21 kg mol; PDI = 1.09), PS homopolymer (*M*_n_ = 1.1 kg mol; PDI = 1.20) and PMMA homopolymer (*M*_n_ = 1.1 kg mol; PDI = 1.20) were obtained from Polymer Source and used as received. Three solutions (67 kg mol^−1^ PS-*b*-PMMA, 1.1 kg mol^−1^ PS, and 1.1 kg mol^−1^ PMMA) of 1% (w/w) in PGMEA were prepared and filtered through a 0.2 micron polytetrafluoroethylene (PTFE) filter.

### Electrospray deposition

The general instrument setup and operation for ESD has been described in a previous paper.^49^ Each solution was drawn into a 1 mL syringe (Hamilton Gastight Syringe Model 1001 TLL, Luer Lock), which were then placed into one of the three gradient syringe pumps connected through polytetrafluoroethylene tubing (1/75′′ ID Cole-Parmer) to a cross junction (IDEX MicroCross) that fed into the spray nozzle. The electrospray mode was monitored by a CCD camera and an automated feedback control algorithm ensured a steady Taylor cone-jet mode by varying the supplied voltage on the spray nozzle. The ESD instrument was set to advance the substrate position while gradually changing the composition of the three solution streams to the spraying nozzle, keeping a constant total flow rate of 10 μl min^−1^. The substrate motor speed was set to 0.02 mm s^−1^ and the substrate temperature was kept at 100 °C. The as-deposited samples were subsequently annealed 12 hours under vacuum at an oven temperature of 220 °C. An error caused a double coating for the stripes with PS : PMMA homopolymer ratios of 62.5 : 37.5 and 75 : 25. Therefore, data for these stripes in Fig. 2 was supplemented with data from the same stripes on another sample with the same polymers deposited at a constant substrate temperature of 190 °C, with no additional vacuum annealing.

The same factors that engender a low viscosity for the homopolymers discussed earlier also instill them with high diffusivity. The combination of high homopolymer diffusivity, high annealing temperature, and a long annealing time may be expected to cause significant deviations from the linear composition profile dictated by ESD as a result of polymer diffusion. Additional analysis indicates this is not the case, however. Specifically, by calculating the transient composition profile as a solution to Fick's second law with an interdiffusion coefficient for the homopolymer estimated based on relevant literature sources,^68,70,72–75^ we determined that the expected deviation in homopolymer mass fractions from values assigned by assuming an ideal linear composition gradient is small enough that it will not significantly impact the broad conclusions of this work. Details of this calculation are provided in the ESI (Section S2).†

### Grazing-incidence small-angle X-ray scattering (GISAXS)

The 11-BM Complex Materials Scattering (CMS) beamline at the National Synchrotron Light Source II was used to acquire GISAXS measurements at increments of 0.2 mm along the 5 mm gradients of increasing homopolymer fraction. An additional 0.5 mm of measurements were included before and after the 5 mm gradient stripe to allow for any misalignment in the sample on the GISAXS stage, yielding a total of 30 X-ray measurements. Conversion of sample position to homopolymer fraction was based on its 100% variation across the 5 mm stripe width. Various position corrections were applied to account for sample misalignment on the GISAXS stage. Specifically, a −0.6 mm correction was applied to stripes up to a 50 : 50 PS : PMMA homopolymer ratio (before the double coating error; see “Electrospray deposition” subsection), while a −0.1 mm correction was applied to the final two stripes corresponding to PS : PMMA homopolymer ratios of 87.5 : 12.5 and 100 : 0 (after the double coating error). The values of these position corrections were determined by minimizing oscillatory variation in the observed ODT compositions. No position correction was determined to be necessary for the ESI data† acquired from films deposited at 190 °C.

Two-dimensional scattering images were collected using a photon-counting area detector (Dectris Pilatus 2 M) placed 5.038 m from the sample. Samples were measured using an X-ray beam of 13.5 keV (*λ* = 0.0918 nm). GISAXS data were collected using a 10 s integration time and a grazing-incidence angle of 0.10°. Silver behenate (AgBH) powder was used as a standard for data conversion to *q*-space. The X-ray beam width was approximately 200 μm in the horizontal direction.

Analyses of GISAXS data were performed using the SciAnalysis toolbox [https://github.com/CFN-softbio/SciAnalysis]. Structural information was extracted from two-dimensional GISAXS scattering images by taking a line cut along the in-plane direction (*q*_*x*_, scattering vector normal to the plane-of-incidence) through the first-order scattering peak at *q*_*z*_ = 0.026 Å^−1^ with an integration window of 0.006 Å^−1^. The peak center position (*q*_*x*,*c*_), width (Δ*q*) and integrated intensity were determined using a Gaussian fitting function with a power-law background to account for diffuse scattering. Line cuts were also compiled in logarithmic waterfall and colormap plots of intensity *vs. q*_*x*_ along the 5 mm samples.

### Electron microscopy

For scanning electron microscopy, PMMA domains within the sample were infiltrated with alumina *via* vapor phase infiltration as described previously.^21,22^ Briefly, infiltration was performed using 4 cycles of exposure to trimethylaluminum and water vapor (300 s each) at 85 °C in a commercial atomic layer deposition tool (Cambridge Ultratech Savannah S100) with a base pressure of < 3 torr. Samples were cleaved for cross-sectional imaging and then a portion of the polymer was removed by O_2_ plasma ashing (March Plasma CS1701F, 100 mTorr, 20 W, 60 s) to reveal alumina replicas of the self-assembled structure. The alumina infiltration extends < 100 nm deep within the films, so PMMA domains closer to the substrate surface appear dark as a result of faster PMMA etching by O_2_ plasma ashing in comparison to PS. Imaging was performed using a Hitachi S-4800 scanning electron microscope at a 5 kV accelerating voltage.

## Conclusions

In conclusion, we demonstrate here a new method for combinatorial investigation of solution-deposited nanomaterial ternary blends based on electrospray deposition of gradient blend films and their characterization with high-throughput GISAXS. Using this method, we map out a morphology diagram for a ternary blend of low molar mass PS and PMMA wet brush homopolymers with a cylinder-forming PS-*b*-PMMA BCP with more than 220 distinct measurements across the entire composition space. Detailed analysis identifies regions of the composition space exhibiting not only the cylinder morphology native to the BCP, but also a disordered phase and highly ordered lamellar and spherical morphologies. The combinatorial samples described in this report were deliberately constructed to produce BCP-homopolymer gradients at various PS : PMMA homopolymer ratios in order to probe trends with respect to overall homopolymer fraction; quantitative analysis reveals systematic dependencies among the nanoscale domain period or degree of order and homopolymer mass fraction and PS : PMMA ratio.

The time necessary to construct a morphology diagram using this method is approximately a couple days, including only a few hours each for film deposition and GISAXS measurements, signifying a remarkable acceleration in comparison to traditional sample-by-sample methods. Even faster measurement and smaller composition increments would be possible (for samples with the same size) when using high-flux micro-focusing beamlines. Moreover, a high degree of flexibility is possible with respect to sample preparation and characterization using this method. For example, one flow rate can be held constant, while imposing a gradient on the other two flow rates (as depicted schematically in ESI Fig. S1†). Alternatively, the influence of film thickness can be probed alongside composition by varying the stage speed or total flow rate during deposition, or by progressively diluting the sprayed solutions with additional solvent. Beyond self-assembly, this method may be adapted to systematically characterize other functional soft and composite material systems where prescribed blending is of paramount importance, such as organic and hybrid photovoltaics,^76–78^ light emitting diodes,^79,80^ and gas separating membranes.^81^ Altogether, the richness of film composition data made rapidly accessible through this method will help accelerate research into a wide variety of nanomaterial blends, especially if paired with autonomous experimentation to explore and analyze large experimental parameter spaces without human intervention.^50,82^

## Conflicts of interest

There are no conflicts to declare.

## Author contributions

K. T. and G. S. D. prepared samples, performed measurements, and analyzed the results. G. S. D. developed the interpretation of the data. K. G. Y. assisted with GISAXS experiments. The manuscript was written through contributions of all authors. All authors have given approval of the final version of the manuscript.

## Supplementary Material

RA-010-D0RA08491C-s001
