# Supplementary material for: High-throughput morphology mapping of self-assembling ternary polymer blends
Source: RSC Adv. 2020 Nov 24;10(69):42529–41. doi: 10.1039/d0ra08491c (PMC9057993; doi:10.1039/d0ra08491c)
Supplement: RA-010-D0RA08491C-s001 [file RA-010-D0RA08491C-s001.pdf]

Electronic Supplementary Material (ESI) for RSC Advances.
This journal is © The Royal Society of Chemistry 2020

Electronic Supporting Information for: High-Throughput Morphology Mapping of Self-Assembling Ternary Polymer Blends

Section S1: Supporting Figures

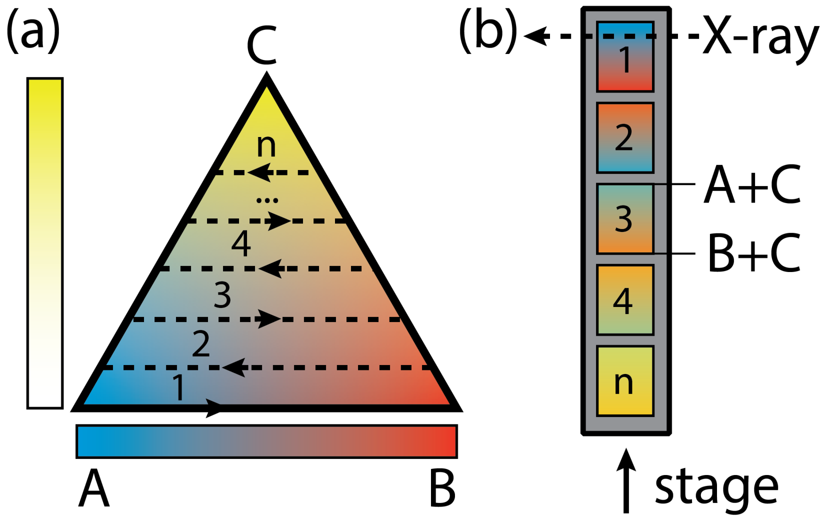

Figure S1: An alternative discretization of a ternary diagram through "n" gradient stripes in which the fraction of A and B vary continuously while holding the fraction of C constant within each stripe. This format is more useful when the transition of interest occurs in the A to B regime since the addition of component C is by specified increments.

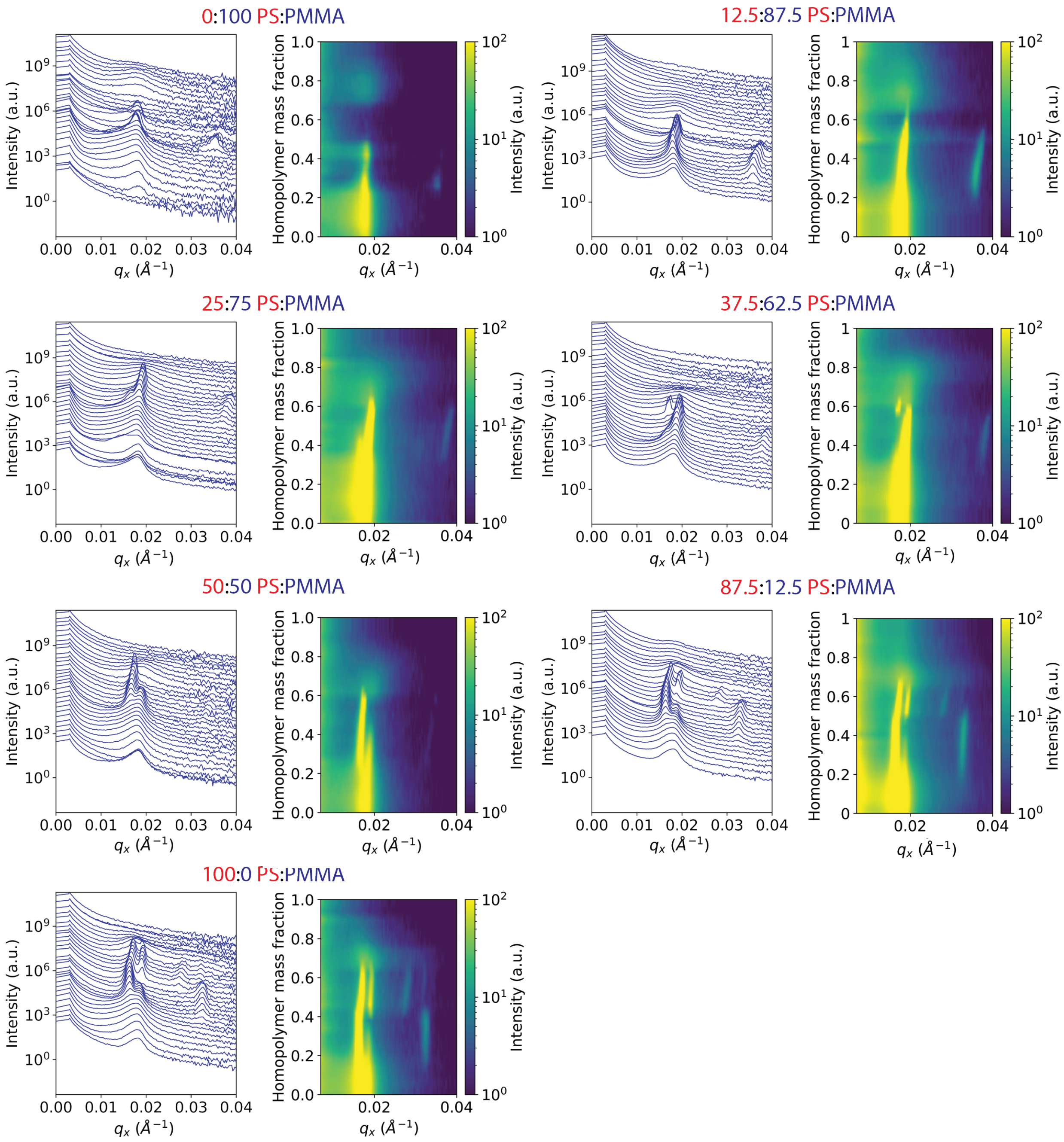

Figure S2: Waterfall and colormap plots compiled from GISAXS q_x line cuts taken over the gradient samples of 67 kg/mol polystyrene-block-poly(methyl methacrylate) (PS-b-PMMA) blended with varying compositions of 1 kg/mol PS and PMMA, respectively. Films were deposited by electrospray deposition at 100°C, followed by post-process oven annealing in vacuum at 220°C for 12 hours. Each pair of waterfall/colormap plots corresponds to a single

stripe on the sample. Composition of 62.5:37.5 and 75:25 PS:PMMA suffered misprinting due to a coding malfunction, so the gradients were supplemented by a later deposition dataset (See Figure SY). However, since the homopolymer blend ratio closely resembled the native block copolymer ratio, the only transitions were disorder to cylinder morphology.

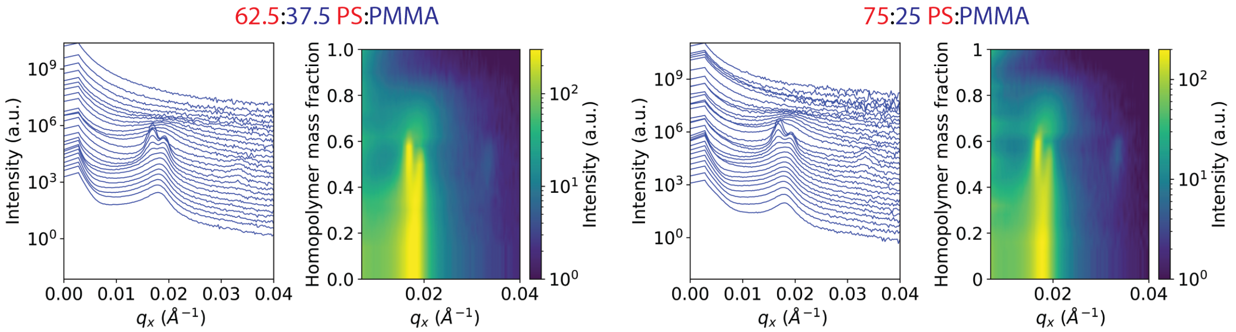

Figure S3: Waterfall and colormap plots compiled from GISAXS q_x line cuts taken over the gradient samples of 67 kg/mol PS-b-PMMA blended with 1 kg/mol PS and 1 kg/mol PMMA at PS:PMMA ratios of 62.5:37.5 and 75:25. Films were deposited by electrospray deposition at 190°C, without further annealing. These stripes were used to make up for missing data points in primary data set deposition at 100°C.

Section S2: Estimated Deviation of the Hompolymer Composition Gradient from Linearity

As noted in the main text, the combination of high homopolymer diffusion coefficients, a long annealing time after electrospray deposition (ESD), and a high annealing temperature may lead to deviations in homopolymer mass fraction from the ideal linear composition gradient across each stripe on the sample as dictated by the ESD protocol. Here we calculate the transient homopolymer composition profile and calculate diffusion coefficients based on literature data to estimate to what extent the composition profile may deviate from a linear gradient.

We consider a stripe of length L_s in the x-direction with a normalized homopolymer mass concentration $\phi_H = C_H/C_{H,max}$ (note $C_{H,max}$ is a constant determined at time = 0). Initially a linear concentration gradient is present across the stripe such that $\phi_H = 0$ at $x = 0$ and $\phi_H = 1$ at $x = L_s$. Thus ϕ_H is effectively equivalent to the homopolymer mass fraction. The composition is then given as a function of position along the stripe (x) and annealing time (t) by Fick's second law in one dimension:

$$\frac{\partial \phi_H(x,t)}{\partial t} = D\frac{\partial^2 \phi_H(x,t)}{\partial x^2} \qquad (1)$$

In the above expression, D is the interdiffusion coefficient. It should be noted that Equation (1) is strictly valid for self-diffusion since D depends on composition, as will be discussed below. However, a solution to equation (1) is still useful for estimating composition profiles in situations where diffusivity is maximized or minimized.

Boundary conditions are given by the fact that no homopolymer diffuses beyond the stripe boundaries:

$$\frac{\partial \phi_H(0,t)}{\partial x} = 0 \qquad (2)$$

$$\frac{\partial \phi_H(L_s,t)}{\partial x} = 0 \qquad (3)$$

The initial condition is a linear composition gradient:

$$\phi_H(x,0) = x/L_s \qquad (4)$$

Equation (1) may then be solved analytically by separation of variables using the above initial condition and boundary conditions to give the following relation:

$$\phi_H(x,t) = 0.5 + \left(\frac{2}{\pi^2}\right)\sum_{n=1}^{\infty}\frac{((-1)^n-1)}{n^2}\cos\left(\frac{n\pi x}{L_s}\right)e^{-\left(\frac{n\pi}{L_s}\right)^2 Dt} \quad (5)$$

The composition profile will be mainly determined by the magnitude of D, where larger values will increase the deviation from a linear gradient. Since the homopolymer is far more mobile than the block copolymer (BCP), it is appropriate to limit attention to homopolymer diffusion. Nevertheless, D is highly composition dependent. To estimate it, we use the model for interdiffusion developed by Kramer et al. to account for the entropy of mixing in incompressible polymer blends.[1] In this case D is given by:

$$D(\phi_H) = \left((1-\phi_H)N_H D_H + \phi_H N D_{BCP}\right)\left(\frac{1-\phi_H}{N_H} + \frac{\phi_H}{N}\right) \quad (6)$$

In equation (6), N and N_H are the degrees of polymerization for the BCP and homopolymer, respectively. The derivation and use of this equation invokes two conditions:

1. We only consider polystyrene chains, and treat the BCP as a PS homopolymer of equivalent molecular weight, a condition that most closely parallels the case of the PS/BCP binary blend. This excludes enthalpic terms, and allows the homopolymer mass and volume fraction to be considered equivalent.
2. Homopolymer mass fractions at time zero are used to calculate the magnitude of D.

In equation (6) D_{BCP} and D_H are the tracer diffusion coefficients for the BCP and homopolymer in the limit of infinite dilution by the opposite polymer (i.e. BCP diluted with homopolymer, and homopolymer diluted with BCP), respectively. The homopolymer molecular weight is well below the entanglement molecular weight, so we estimate D_H using the Rouse model[2] giving

$$D_H = \frac{k_B T}{N_H \zeta_{eff}(\phi_H)} \quad (7),$$

where k_B is Boltzmann's constant and T is absolute temperature (in Kelvin). The BCP diluted in an unentangled homopolymer matrix can be considered to diffuse as a Zimm coil[3] with a Rouse matrix viscosity[4] giving,

$$D_{BCP} = \frac{m_0 k_B T}{\pi \rho N_{Av} a^3 \zeta_{eff}(\phi_H)} N^{-1/2} N_H^{-1} \qquad (8)$$

where m_0 is the monomer molecular weight, ρ is the mass density, N_{Av} is Avogadro's number, and a is the monomer segment length (0.6 nm)[5].

An effective monomeric friction factor ($\zeta_{eff}(\phi_H)$) is necessary in equations (7) and (8) because its magnitude depends not only on the diffusing polymer itself, but on the polymer matrix in which it is diffusing.[6–8] Considering only PS homopolymer, differences in ζ are directly attributable to different glass transition temperatures for both the diffusing and matrix polymers. The temperature-dependent magnitude of the PS friction factor (ζ_{PS}) can be estimated based on a Williams-Landel-Ferry expression,[8]

$$\log(\zeta_{PS}) = \log(\zeta_{PS})_{T_g} - a_T(T, T_g) = 0.69 - \frac{12.69(T - T_g)}{85.51 + (T - T_g)} \quad (9),$$

where T_g is the glass transition temperature, $\log(\zeta_{PS})_{T_g}$ is the logarithm ζ_{PS} at T_g, and $a_T(T, T_g)$ is a temperature dependent shift factor. Note that we use the shift factor obtained through fitting experimental data by Milhaupt et al.,[8] but have reduced the value of $\log(\zeta_{PS})_{T_g}$ to better agree with available experimental measurements of the self-diffusion of low molecular weight PS.[9] By lowering the friction factor, this adjustment ultimately increases our estimates for D.

Increased free volume for the very short homopolymers used in this work will substantially depress T_g. This effect can be estimated by the Flory-Fox Equation,[10]

$$T_g = T_{g\infty} - \frac{K}{M_n} \qquad (10),$$

where $T_{g\infty}$ is the maximum T_g at infinite molar mass (effectively a fit parameter), M_n is the number average molar mass, and K is a fitting parameter. To obtain a T_g estimate for the 1.1 kg/mol PS used in this work, we applied Equation (10) to tabular data from Claudy et al.[11] This fit is shown in Figure S4, from which an estimated T_g of 40 °C is obtained for the 1.1 kg/mol PS. Inserting this value into Equation (9) yields a homopolymer friction factor of $\zeta_H = 1.2\times10^{-8}$ dyn-s/cm at the annealing temperature of 220 °C. As noted above, we treat the BCP as a PS homopolymer with a molar mass of 67 kg/mol. Applying Equations (10) and (9) then yields T_g = 100 °C and $\zeta_{BCP} = 1.9\times10^{-7}$ dyn-s/cm, respectively. Note that T_g data for molar masses less than 0.5 kg/mol for Ref. 11 were excluded from fitting as the resultant fit was poor and increased the T_g estimate to ~50 °C, thereby leading to a higher-valued friction factor and a concomitant reduction in D.

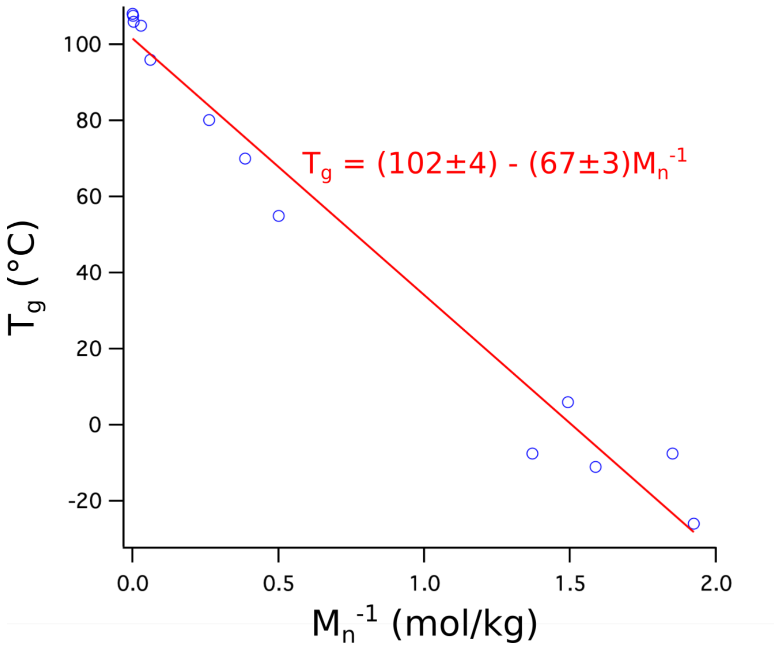

Figure S4: Fit of Flory-Fox equation[10] to glass transition temperature (Tg) data for PS.[11]

Between these limits, the effective friction factor can be estimated using an Arrhenius mixing rule:[8]

$$\log(\zeta_{eff}) = \phi_H \log(\zeta_H) + (1-\phi_H)\log(\zeta_{BCP}) \qquad (11)$$

Combining equations (6)-(8) and (11) results in diffusion coefficients on the order of ~10^{-8} cm^2/s across a broad range of compositions, with a maximum value of $D_{max} = 5.8\times10^{-8}$ cm^2/s at a $\phi_H \approx 0.58$, as shown in Figure S5. D changes surprisingly little for $\phi_H <$ ~0.7 because diffusion is limited by the high friction of the BCP matrix at low homopolymer mass fractions, while D is inhibited by slow BCP counter-diffusion at the homopolymer rich end of the gradient.

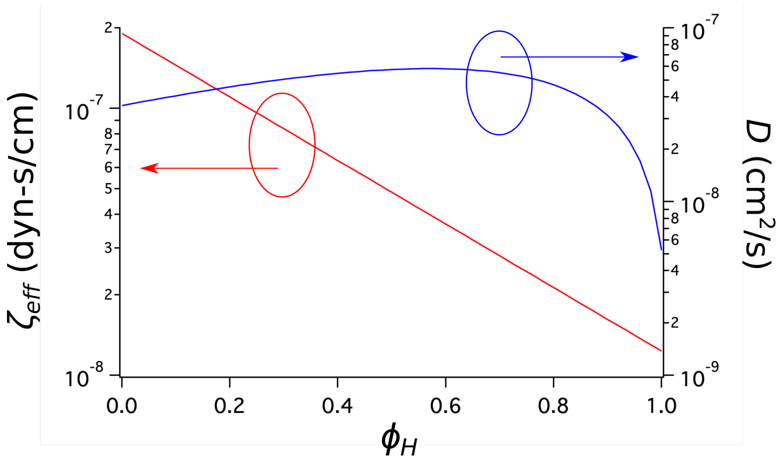

Figure S5: Calculated values for the effective friction factor (ζ_{eff}) and the interdiffusion coefficient (D) as functions of the homopolymer mass fraction (ϕ_H).

The composition profile calculated using Equation (5) (truncated to $n = 20$) with $D = D_{max}$ after 12 hours of annealing at 220 °C is shown in Figure S6a. Also included are the calculated composition profiles at $t = 0$ (which does not depend on D), and after annealing with $D = D_{max}/10$. Deviations from a linear composition profile are only apparent near edges of the stripe,

indicating that this deviation is negligible for x in the range of 1.0 to 4.0 mm, corresponding to homopolymer mass fractions in the range of 0.2 to 0.8.

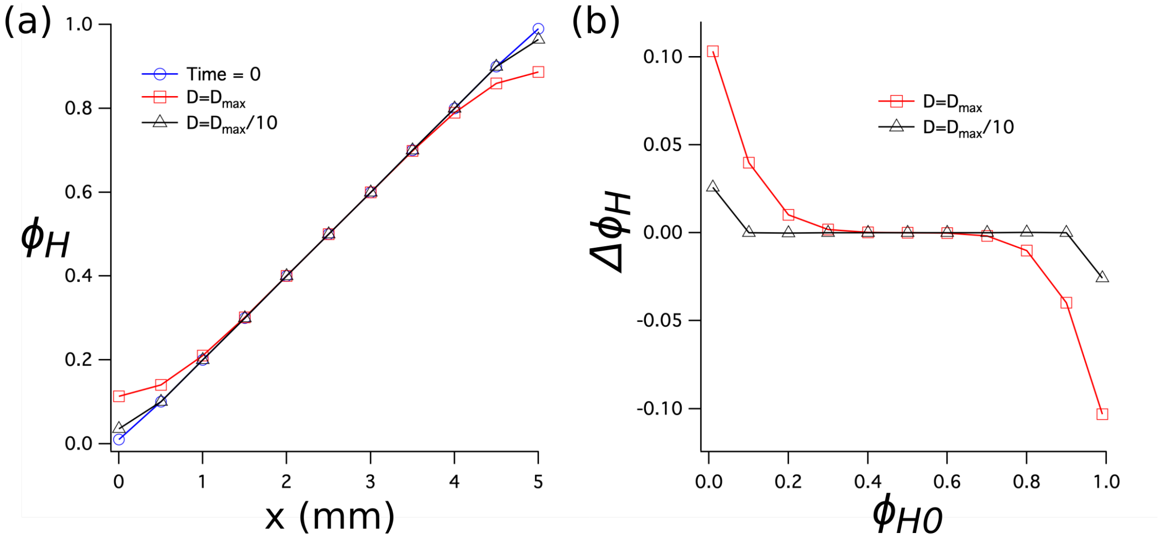

Figure S6: Calculated composition profiles using equation (5) as discussed in the text. (a) Homopolymer fraction (ϕ_H) versus stripe position (x). Included are the calculated initial composition, the composition after annealing for 12 hours at 220 °C assuming a maximum estimate for the interdiffusion coefficient (D_{max} = 4.0×10^{-8} cm^2/s), and the composition after the same annealing using $D = D_{max}$ /10. (b) Composition deviation from its initial value ($\Delta\phi$) versus the initial composition (ϕ_{H0}) for both post-annealing cases in (a).

To better visualize the magnitude of the composition deviation ($\Delta\phi_H = \phi_H(x,t) - \phi_H(x,0)$), we plot it against $\phi_{H0} = \phi_H(x,0)$ in Figure S6b. Again it is clear that the magnitude of $\Delta\phi_H$ is negligible when the composition is in the range of 0.2 to 0.8 when $D = D_{max}$. In this composition range, the maximum magnitude of $\Delta\phi_H$ is ~0.01. The magnitude of $\Delta\phi_H$ increases to ~0.1 at the ends of the gradient (i.e. the stripe edges). However, the magnitude of D also drops precipitously as $\phi_H \rightarrow 1$, as shown by Figure S5, which suggests a maximum $\Delta\phi_H$ on the order of ~0.03, as indicated by the calculation in which $D = D_{max}/10$.

It is also worth returning to the fact that we have ignored any contribution from PMMA in the preceding analysis. In general, experiments have indicated that PMMA possesses a significantly higher monomeric friction factor than PS[6,8] and generally exhibits a slightly higher glass transition temperature than PS at comparable molar masses.[8] Moreover, adding PMMA will introduce additional enthalpic penalties that inhibit diffusion,[12] especially when considering the periodic energy barriers associated with ordered domain structures.[2] Therefore, including PMMA is expected to only diminish the magnitude of D, perhaps by an order of magnitude or more, and hence further reduce the deviation from a linear composition gradient, $\Delta\phi_H$. The effects of including PMMA would become especially significant in the BCP rich regions of the composition space, and away from the PS/BCP binary blend edge of the ternary composition space. Therefore, we conclude that deviations from a linear composition profile are likely small enough that they do not significantly impact our broader experimental conclusions.

Finally, we comment on the magnitude of the viscosity (η) of the ~1 kg/mol homopolymers relative to the 67 kg/mol BCP. We note that under the Rouse model, $\eta \propto N\zeta$.[8] As noted above, the substantial increase in free volume for the 1.1 kg/mol PS homopolymer lowers its T_g relative to a 67 kg/mol PS homopolymer by approximately 60 °C, which in turn lowers the value of ζ for the 1.1 kg/mol PS by an order of magnitude or more at temperatures of 220 °C or less. Critically, this ratio increases as temperature is reduced. Furthermore, N = 648 for the BCP, while N = 10 for the 1.1 kg/mol PS homopolymer. Taking these parameters into consideration, the ratio of viscosity between the BCP and the homopolymer is ~10^3. This estimate does not account for chain entanglement within the BCP that will increase this ratio even further. Therefore, the homopolymer viscosity is more than three orders of magnitude lower than the viscosity of the BCP.

Section S3: References

1 E. J. Kramer, P. Green and C. J. Palmstrøm, *Polymer (Guildf).*, 1984, **25**, 473–480.

2 H. Yokoyama, *Mater. Sci. Eng. R Reports*, 2006, **53**, 199–248.

3 P. F. Green and E. J. Kramer, *Macromolecules*, 1986, **19**, 1108–1114.

4 D. S. Pearson, L. J. Fetters, W. W. Graessley, G. Ver Strate and E. Von Meerwall, *Macromolecules*, 1994, **27**, 711–719.

5 M. Rubinstein and R. H. Colby, *Polymer Physics*, 2003.

6 P. F. Green, *Macromolecules*, 1995, **28**, 2155–2158.

7 S. F. Tead and E. J. Kramer, *Macromolecules*, 1988, **21**, 1513–1517.

8 J. M. Milhaupt, T. P. Lodge, S. D. Smith and M. W. Hamersky, *Macromolecules*, 2001, **34**, 5561–5570.

9 G. Fleischer, *Colloid Polym. Sci.*, 1987, **265**, 89–95.

10 T. G. Fox and P. J. Flory, *J. Appl. Phys.*, 1950, **21**, 581–591.

11 P. Claudy, J. M. Létoffé, Y. Camberlain and J. P. Pascault, *Polym. Bull.*, 1983, **9**, 208–215.

12 P. F. Green and B. L. Doyle, *Phys. Rev. Lett.*, 1986, **57**, 2407–2410.
